# Waiting for the wave: Political leadership, policy windows, and alcohol policy change in Ireland

**DOI:** 10.1016/j.socscimed.2021.114116

**Published:** 2021-08

**Authors:** Matthew Lesch, Jim McCambridge

**Affiliations:** University of York, York, United Kingdom

**Keywords:** Alcohol, Public health, Multiple streams approach, Political leadership, Ireland, Policy entrepreneurs, Evidence-based policy, Qualitative

## Abstract

Existing research has identified numerous barriers to the adoption of public health policies for alcohol, including the cross-cutting nature of the policy problem and industry influence. Recent developments in Ireland suggest that while formidable, such barriers can be overcome. Ireland's 2018 alcohol legislation adopts key evidence-based measures, introducing pricing, availability and marketing regulations that are world-leading in public health terms. Drawing primarily on the Multiple Streams Approach (MSA), this study investigates the adoption of the Public Health (Alcohol) Act 2018. We draw data from 20 semi-structured interviews with politicians, government advisors, public health experts, and advocates, as well as from relevant primary documents, newspaper articles, and other material in the public domain. We find that increased public attention to alcohol-related harms in Ireland (problem stream), developments within the institutional location of policymaking (the policy stream), and the political pressure exerted by politicians and advocates (the political stream) all combined to open a policy window. Unlike previous alcohol policy reform efforts in Ireland, several personally committed and well-positioned leaders championed policy change. This study suggests that political leadership might be important in understanding why public health approaches to alcohol have been embraced in some contexts but not in others.

## Highlights

• Evidence of policy failure is necessary but insufficient for alcohol policy change.
• Framing alcohol as a public health problem is key to getting the issue prioritised.
• The alcohol industry poses a significant but not insurmountable barrier to reform.
• Skilful political leadership can offset the influence of industry opposition.

## 1 Introduction

Alcohol consumption has long been a source of major health and social problems in Ireland (Mongan and Long, 2016; Mongan et al., 2007). A combination of factors has undermined previous attempts to address alcohol, specifically as a public health issue. First, the alcohol industry wields considerable political and economic power in Ireland (Butler, 2009, 2015; Hope and Butler, 2010; Hope, 2006, 2014; Mercille, 2016; Butler et al., 2017; Calnan et al., 2018). Researchers have identified the liberalisation of the alcohol retail sector in the 2000s and the industry's success in thwarting new regulations as key indicative examples (Mercille, 2016). A second barrier has been the failure of the government to develop a “fully integrated approach” across its several departments and agencies (Hope, 2006). In Ireland, about 11 different departments, ranging from health to finance, possess some responsibility for alcohol-related issues (Hope, 2006). The cross-cutting nature of alcohol has made coming to grips with this issue challenging.

This present study considers how such barriers have been overcome in passing alcohol public health legislation after public health ideas on alcohol first gained traction. In 2009, the government commissioned a steering group to study alcohol-related harms, and how they may be reduced (Department of Health, 2012). The report's recommendations formed the basis of the landmark Public Health (Alcohol) Bill (Government of Ireland, 2015). Between 2013 and 2018, the bill encountered multiple controversies and faced intense political scrutiny, first within government, and subsequently in the Oireachtas (parliament). The legislation was adopted in October 2018, the first time Ireland legislated alcohol as a public health issue (Lesch and McCambridge, 2021a). Its content comprehensively adopts key evidence-based measures, and it is world-leading in public health terms in addressing barriers arising from the cross-cutting nature of the policy problem (Baggott, 2010). The other key barrier is alcohol industry involvement in policymaking, which exists widely elsewhere, particularly in other major alcohol producer countries (Holden et al., 2012; Lesch and McCambridge, 2020, 2021b; McCambridge et al., 2013, 2014, 2018; Katikireddi et al., 2014a).

The multiple streams approach (MSA) is primarily used to explain why governments take up certain issues at a particular time (Kingdon, 1995; Zahariadis, 2003). Policy entrepreneurs are key in this conceptualisation of the policy process. These are the actors who leverage their policy knowledge and political insights to advance specific policy solutions (Kingdon, 1995). Windows of opportunity open up for policy entrepreneurs when three independent streams converge: 1) the problem stream; 2) the policy stream and 3) the politics stream. The *problem stream* refers to the set of issues or conditions that become perceived as problematic. Policy actors' attention to these problems emerge from changes in indicators, focusing events, and policy feedback (Kingdon, 1995; Zahariadis, 2003). The *policy stream* encompasses specific solutions that have been identified for addressing a given problem. Ideas in the policy stream tend to be generated within specialised policy communities (Kingdon, 1995). Finally, the *political stream* refers to how shifts in the national mood, interest group environment, or decision-making personnel can influence what is considered politically acceptable (Kingdon, 1995; Zahariadis, 2003). Policy entrepreneurs’ success hinges on “waiting for the big wave”, the precise moments when these streams can be fruitfully combined (Zahariadis, 2003).

The MSA has been previously applied to alcohol policy in various studies (Butler, 2015; Butler et al., 2017; Nicholls and Greenaway, 2015; Katikireddi et al., 2014b; Hawkins and McCambridge, 2019). In previous applications to Ireland, researchers have identified the political stream as a key barrier to policy change (Butler, 2015; Butler et al., 2017). Despite indications of policy failure, politicians have often lacked the political will to carry out meaningful policy change. Thus recent developments in Ireland, and specifically the enactment of the Public Health (Alcohol) Bill in 2018, offer an opportunity to assess how the political stream has shifted over time.

The MSA represents one way to conceptualise policy change. An alternative approach is the Advocacy Coalition Framework (ACF) (Weible et al., 2009). The ACF highlights the role of collective action, specifically advocacy coalitions, in generating policy change. This framework considers policy actors, including interest groups, experts, and government officials, to be aggregated into competing coalitions. At the core of each coalition is a shared belief system; actors possess similar normative ideas about how an issue should be defined and how problems should be addressed. In the ACF, policy-oriented learning or external shocks are the main sources of policy change (Weible et al., 2009).

The ACF has been applied to recent alcohol policy developments in Scotland, England and Ireland, with public health actors and the alcohol industry identified as opposing coalitions in these contexts (Lesch and McCambridge, 2021b; Fergie et al., 2019; Thom et al., 2016). According to these studies, public health actors conceptualise alcohol consumption as a population-level problem and thus advocate for stricter laws that limit the availability, affordability and promotion of alcohol. In contrast, the industry coalition claims excessive alcohol consumption only affects a minority of drinkers, and thus regard targeted approaches as more appropriate policy solutions (McCambridge et al., 2013; Fergie et al., 2019). The ACF's analytical strength is it helps researchers understand why coalitions form and how they stick together. The framework provides less guidance in explaining how decision-makers respond to their tactics, and why the effectiveness of these tactics varies over time. To that end, the MSA serves a useful complementary function. By linking agenda-setting to broader shifts in the political environment (i.e., policy windows), the MSA clarifies the conditions for policy change. Thus, while the main focus of this article is on applying MSA, we also draw on concepts from the ACF to explore the role of advocacy coalitions.

## 2 Methods

This article explores how the Public Health (Alcohol) Bill 2015 made its way on to the agenda, and how the legislation was subsequently formulated. We use within-case analysis (Collier et al., 2010) to construct a record of the policy's development, focusing specifically on the period between 2008 and 2018.

Drawing on primary government policy documents, news articles, and secondary sources, the study charts the sequencing of developments within the policy process. We gathered primary documents by conducting online searches of government websites. The Nexis database was used to access relevant media published between 2012 and 2018 in three major national newspapers: *Irish Times, Irish Examiner* and *Irish Independent*.

Semi-structured interviews were conducted with 20 individuals, including public health advocates and leading medical practitioners (13), public health experts (2), politicians (4) and a policy advisor (1). Alcohol industry representatives were not included as part of the interview sample, as access may be challenging and resulting data complex to interpret. To examine the views and activities of the alcohol industry we consulted a range of sources, including newspaper articles, press releases, and lobbying registry data. The main purpose of this study was to investigate how the alcohol bill got on the agenda and was subsequently passed. This makes both advocacy coalitions relevant to the analysis but not the central focus.

We conducted interviews in-person or via Zoom between September 2019 and August 2020. Interviewees were purposively sampled after having been identified through a combination of government documents, media reporting, and snowball sampling. Interviewees were selected because they had either been active participants in the policy process or possessed in-depth knowledge of the evolution of alcohol policy in Ireland. E-mail recruitment yielded a response rate of ~55% of all those targeted. We provided participants with a two-page information sheet that described the purpose of the study and the rationale for recruitment. All participants provided informed consent prior to their interview. Ethics approval for the study was provided by the Research Governance Committee at the University of York in February 2019.

The first author undertook all interviews. The interviews were recorded with permission and then transcribed verbatim. Transcripts were initially thematically coded and analysed using NVivo 12 by the first author. The transcripts were subsequently analysed in an iterative manner, with both authors reviewing them, generating and refining thematic material, and agreeing on interpretation.

A broad set of themes, informed by policy studies research, were initially developed to organise and analyse the data. These included codes for different types of actors (e.g., advocates, the alcohol industry and politicians), different stages of the policy process (e.g., agenda-setting, policy formulation, and implementation) and different causal mechanisms (e.g. issue framing, coalition-building, and policy feedback). The initial thematic analysis revealed the importance of policy entrepreneurs and issue framing in driving the momentum of policy development. As such, we developed specific codes associated with the MSA (e.g., problem stream, policy stream and politics stream) and its core concepts (e.g., policy entrepreneurs, focusing events) to analyse the data in moving towards the refinement of thematic content. Below we present a chronologically organised account of the context and the developments within each of the three streams that led to the opening of the policy window, and the resulting decision-making steps leading to the adoption of the legislation.

We took various steps to ensure the reliability of the interview findings. Descriptions of key events and decisions were cross-referenced with other data sources, including public statements, primary documents and newspaper coverage, as part of the wider process of triangulation in generating secure inferences. This necessarily addressed the interpretations of the significance of particular issues by the interviewees themselves.

A potential limitation is that some of the key actors involved in decision-making were not included as part of the interview sample. Our efforts to recruit two of the health ministers were unsuccessful. Access to elected officials poses a common challenge for researchers probing politically sensitive issues. One potential solution is to recruit participants who can share insights into the actors and/or issues of interest but who are no longer active in politics or industry. A second solution is to interview gatekeepers such as public officials and political staff who may be “privy to behind-the-scenes action” (Marland and Esselment, 2018). We implemented both strategies with some success. In the case of industry actors, we relied on secondary data sources to better understand their policy preferences and strategies, as well as the accounts provided by the interviewees.

## 3 Results

### 3.1 Policy context

As in other major producer countries, the public health community and the alcohol industry have formed two opposing coalitions in Ireland. The former is made up of advocacy organisations, civil society groups, health experts, medical professionals and government officials (primarily in the Department of Health). The core members of this coalition – Alcohol Action Ireland, the Royal College of Physicians Ireland (RCPI) and other civil society groups – have organised under the umbrella of the Alcohol Health Alliance Ireland (Lesch and McCambridge, 2021b). The alcohol industry coalition consists of producers, trade associations, wholesalers and retailers, as well as allies in other sectors and non-health government departments. Industry actors have coordinated their political activities through industry associations. This has occurred primarily through Drinks Ireland, formerly the Alcohol Beverage Federation of Ireland (ABFI), a drinks sector association that operates within IBEC, the main business lobby group in Ireland (Interview C-3, Interview C1). Although alcohol industry actors form the core of the coalition, they have secured support from other actors and sectors when common interests have been identified in policy debates (see below).

Both coalitions have competed to influence the direction of alcohol policy in Ireland. Public health actors have historically found it difficult to counter the alcohol industry's influence within successive Irish governments (Interviews A-4, A-5, B-1). Ireland's rising prosperity in recent decades has meant that economic priorities have dominated public health issues in discussions over alcohol policy. For example, in November 2000, the government allowed licensed establishments to stay open for longer when it adopted the *Intoxicating Liquor Act 2000*. At the time, the Justice Minister also established a Commission on Liquor to review Ireland's alcohol licensing system. The Commission produced several reports between May 2001 and April 2003 but failed to engage with the concerns identified previously. As one former policy advisor in the Department of Health explained, the government relied on a self-regulation system for the alcohol industry. Health officials were reportedly less comfortable with this approach, deeming self-regulation as an ineffective response to alcohol-related harms (Interview D-1).

The Department of Health's inability to establish a new approach to alcohol reflected how influential the industry coalition's framing of ideas had been within government. As one analyst explained, Ireland has traditionally had “two parallel and conflicting alcohol policy processes”, one concerned with “exploring the extent to which the drinks trade could be deregulated” and the other identifying “regulatory systems” for reducing consumption and promoting public health (Butler et al., 2017).

Over the past 15 years, however, the public health coalition has steadily gained more influence, helping shift the debate over alcohol. In 2006, a parliamentary committee suggested that the lack of a permanent policy structure for alcohol, unlike drugs, was a key barrier to addressing public health concerns (Committee on Arts, Sport, Tourism, Community, Rural and Gaeltacht Affairs, 2006). Furthermore, in 2008, during the National Drug Strategy steering group's public consultations, concerns about alcohol consumption were “widespread” across local communities (Department of Community, Rural and Gaeltacht Affairs, 2009). Several interviewees articulated the level of public concern as a key driver of change (Interviews A-5, A-6, and A-7). According to one advocate:

When [government officials] went out to consult communities around the drug strategy, [the community] said ‘look, yeah, we do have issues around drugs but the bigger problem is alcohol’ (Interview A-2).

Growing dissatisfaction with the status quo, particularly at the local level, presented a key justification for health officials to assert more control over alcohol policy. Experts and senior health officials recognised that addressing public concerns and better establishing public health influence required a different governance strategy. They also recognised that legislation was necessary for such an approach. As one public health expert recalled:

No matter how many strategic task forces you bring together [or] how much you put the evidence together … at the end of the day, legislation trumps policy … comprehensive [change] … just wasn't going to happen … without legislation (Interview B-1).

From December 2008, Dr Tony Holohan was the department's new Chief Medical Officer (CMO). Holohan wanted “a more permanent institutional structure for alcohol” and reinforced the parliamentary committee's recommendation to incorporate alcohol into the retitled National Substance Misuse Strategy (NSMS) (Interview D-1). The establishment of an alcohol steering group, with Holohan as co-chair, charted a new direction for alcohol policy and strengthened the institutional position of the public health coalition.

### 3.2 Problem stream

The main motivation for placing alcohol under the NSMS remit related to the increased visibility of alcohol-related harms. In the 1990s and 2000s, significant increases in alcohol consumption led to increased harms (Hope and Butler, 2010), and recorded per capita consumption approximately trebled between 1960 and 2001 (Organisation for Economic Cooperation, 2011). As one former policy advisor recalled:

[By 2009] alcohol consumption was over 14 L per capita … it was just completely out of control … the government became aware of that … [and] the public health community began to say, ‘listen, we have to get control of this’ (Interview D-1).

In the late-2000s, there also was growing recognition across the medical profession, particularly among liver specialists, that the long-term consumption trends were feeding through to alcohol-related harms. As one interviewee from the RCPI recalled:

Our [doctors] had been going to the international meetings of liver specialists and were alarmed by the rate of liver cirrhosis in Ireland … they were concerned by the profile of the people who were being affected … there were more women [and] younger people … [Liver disease] was something that they had traditionally seen in older men (Interview A-3).

The nature of the issue, however, had not been immediately obvious to the government. As one public health expert explained:

There was this lag between the consumption and the harms, but the harms came out really strongly … By the time we came to the mid-2000s, a lot of the legacy of [increased consumption] was coming through … That's when the healthcare providers and the social care providers started saying [to the government], ‘this is under our door‘(Interview B-2).

Despite the chorus of concerns from doctors and other experts, the Department of Health did not possess the data to validate or further investigate such claims. Few studies in Ireland tracked alcohol consumption or its impact on public health in Ireland (Interviews B-1, B-2 and A-4). In response, the Health Research Board (HRB) began undertaking such research, combing through existing data sources, including the National Drug-Related Deaths Index, National Drug Treatment Reporting System, and consumption data from the Central Statistics Office (Interview B-2). According to one expert, the HRB's findings “got a huge reaction” from the media and civil society.” (Interview B-2). As one policy advisor explained, the research provided “new information, insight, [and] knowledge” about alcohol's impact at a “crucial time.” (Interview D-1).

One of the reasons that the HRB's research was so valuable is because it enabled advocates to link alcohol-related harms to broader problems with the health system, thereby mainstreaming alcohol as a health policy issue. During this time, concerns about the health service in Ireland had become “a hot political issue” (Interview A-9). Media attention to the “trolley crisis” in Ireland generated a key opportunity for those advocating a public health approach to alcohol (Interview A-4). Advocates and public health experts responded by drawing explicit links between the harms documented in the HRB research and the fiscal pressures on the health system (Interview C-4). According to Ged Nash, a former Minister of State, and Labour TD (member of parliament):

The evidence and data … echoe[d] conversations with health professionals … The doctors talk[ed] about the absolute catastrophe on a Friday, Saturday and Sunday … People coming in with alcohol-related injuries … [Health care professionals] felt very strong about it … saying ‘look, we really need to change our approach here’ (Interview C-1).

Concerns about alcohol, and specifically “access to cheap drink”, were also increasingly being raised during politicians' clinics with their constituents (Interviews C-1 and C-2). As Alex White, also a Labour minister, explained, although there was pressure coming from “multiple sources”, the public's desire to “have something done [about alcohol] should not be underestimated” (Interview C-2).

Advocates working at the local level also stressed the importance of public pressure. Politicians in Ireland “have a very sensitive ear for what's moving at the grassroots level” and so they would have been quite reluctant to “dismiss opinions” coming from their constituents (Interviews A-5, A-6 and A-7). Alcohol thus had become an unignorable problem for Irish public health and society.

### 3.3 The policy stream

When the government decided to integrate alcohol and drugs into a combined NSMS, it established the steering group to help formulate the alcohol part. The steering group was highly diverse, comprising officials across government departments (e.g., health, justice, transport, tourism and sport), major health NGOs, civil society groups, and representatives from the alcohol industry. Its task was to specify measures that could “tackle the harm caused to individuals and society by alcohol use and misuse” (Department of Health, 2012). The group met several times between December 2009 and November 2011. Stakeholder submissions, as well as reports and policy documents produced at the national and international level, informed the group's discussions (Interviews A-8 and B-2). The steering group developed and presented its findings and recommendations “in the early days of a new government” (Interview A-8).

The group's final report, released in February 2012, recognised alcohol as a major societal problem. It argued that the government had a “crucial role by intervening to prevent problems” and identified “price, availability and marketing” as the key drivers of alcohol consumption (Department of Health, 2012). The report thus adopted a public health perspective that sought to address the environmental determinants of health bearing upon the key proximal behavioural risk factor of alcohol consumption. In its chapter on supply, the report offered several key evidence-based population-level measures for decreasing consumption, including (but not limited to) measures to influence the availability, marketing and price of alcohol (See Box 1).

Box 1 **Selected Recommendations from the Steering Group's 2012 Report**

**Table:** 

Policy Lever	Description
**Price**	Introducting a legislative basis for minimum unit pricing
**Availability**	Requiring structural separation of alcohol from other products in supermarkets and other mixed-retail outlets
**Marketing**	Numerous restrictions on alcohol marketing and advertising, including: • 9:00 p.m. watershed for alcohol advertising on traditional media • Restricting alcohol advertising in cinemas to films classified as being suitable for over-18s • Subjecting all alcohol advertising in print media to stringent codes, with independent monitoring • Prohibiting outdoor alcohol advertising • Phasing-out drinks industry sponsorship of sport and other large public events

Alt-text: Box 1

Box 2 **Summary of Findings**

**Table:** 

Development	Key Actor(s)	Evidence
Problem stream	Experts • Department of Health • Health Research Board • Royal College of Physicians Ireland	Perception that alcohol consumption was entrenched at levels which were internationally high, with major health harms associated with them.
Policy stream	Steering Group • CMO, Dr Tony Holohan • Department of Health	Identified clear set of population-level policy alternatives for the government from the international evidence-base.
Political stream	Health ministers • Various junior health ministers • Leo Varadkar Advocates • Dr Frank Murray • Alcohol Health Alliance Ireland	Election results and efforts by the public health advocacy community persuaded signalled a shift in the public mood. Advocates detected shifts in the public mood and mobilised experts and organisations.

Alt-text: Box 2

Alcohol industry representatives refused to endorse the report and released two minority reports in protest (Alcohol Beverage Federation of Ireland, 2011; Mature Enjoyment of Alcohol in Society Limited, 2011). During the steering group's deliberations, industry actors continuously sought to re-frame the policy problem. As one expert from the steering group recalled:

We argued [with the alcohol industry representatives] about the terms of reference and the language around alcohol use and misuse … We were still on meeting three discussing this and really that just shows how important language is. They really fought against having the term alcohol use used [and] always pushing [us] towards misuse (Interview B-2).

This distinction between use and misuse was crucial. If alcohol use throughout the population was understood to the source of the social and public health problem, then policy measures to reduce overall use were needed (Babor et al., 2010). If, however, the misuse of a minority of heavy drinkers or “alcoholics” was the problem, there was no need for measures that increased price and reduced availability and marketing. The key contribution of the international research literature drawn upon by the public health coalition was that reduction in overall drinking was needed for harms to be reduced across society because they were so closely related at a population level (Babor et al., 2010). When discussions moved to particular policy measures, industry actors found some support for their concerns. Officials from the Departments of Transport, Tourism and Sport (TTS) and Arts, Heritage and the Gaeltacht held that economic impacts of sponsorship restrictions needed to be considered. These officials eventually backed the majority's recommendation but the episode revealed the capacity of the industry to form coalitions on an issue-by-issue basis.

The steering group's comprehensive review of the international and national evidence, its broad membership, and its concrete set of policy recommendations set it apart from earlier institutional processes. The chair, the CMO Holohan, could also use his institutional position to ensure that the minister gave proper consideration to the report. As one steering group member recalled:

A lot of [the public health bill] emanate[d] from [the steering group] … that's why the industry [was] quite worried about [the process] because when you have a body that's chaired by the Chief Medical Officer and [it] has looked at all the evidence and had all the discussions and then comes up with those recommendations, that's a good platform to start with (Interview A-8).

Despite evidence of a clear policy problem and the availability of evidence-informed policy responses, the government's willingness to act on these recommendations could not be taken as a given. As another public expert explained:

Evidence is one thing you have in your armament [but] it becomes less useful as the debate goes on … [Policy action] comes down to timing [and the] order of the things on the agenda. It's about who's there. It's about political allegiances. It's about all that kind of stuff (Interview B-1).

In 2012, at the direction of the CMO, the Department of Health commissioned research on public support for the steering group's key policy recommendations. According to one expert, the CMO had asked the HRB to “gauge public support” for stricter alcohol measures. The thinking was that if the government felt it had public backing it could claim “well, the people want it.” The survey research found “majority support” for every recommendation “with the exception of the [ban on] sports sponsorship” (Interview B-2).

### 3.4 The political stream

Between the appointment of the steering group and the release of its recommendations in 2012, the political landscape had shifted profoundly in Ireland. In the 2011 general election, the government, led by Fianna Fail, suffered its worst-ever defeat. Fine Gael won the most seats in parliament and formed a coalition government with Labour, which had won the second-highest number of seats. The shift in political power had implications for the development of alcohol policy. Several government ministers, particularly those from Labour, wanted to act on the recommendations of the steering group. Following the report's release, the junior minister for primary care, Roisin Shortall, began “migrating the conclusions of the study” into legislative measures (Interview C-2). Tensions between Shortall and the government (over the bill and other issues) led to her resignation (Holland, 2012). Shortall's successor, Alex White, a Labour TD continued to push for the implementation of the steering group's recommendations (O’Halloran, 2012). As White recalled:

I did a lot of discussions and negotiations with other ministers in the government cabinet … I was sort of marshalling the thing … and discussing the various elements of it with different ministers, with a view to bringing forward the heads of the Bill to cabinet (Interview C-2).

In June 2013, the media reported “fretting” within the coalition, after the Minister of Health, James Reilly (Fine Gael), and his deputy, Alex White, presented their policy proposal to the cabinet (O'Connell, 2012), largely following the main recommendations of the steering group. The proposal included a plan to ban the alcohol industry from any sports sponsorship. Although the proposal found some support within Labour (Brennan and Kelly, 2013), this part faced immediate pushback from Fine Gael members. Moreover, Diageo, comfortably the largest producer company, threatened to reduce operations in Ireland if the policy progressed (Molloy, 2013).

During 2013, conflicts between the health ministers and their colleagues prominently included Leo Varadkar, the Transport, Tourism and Sport (TTS) minister. Key sporting organisations, including the Gaelic Athletic Association (GAA), the Irish Rugby Football Union (RFU), and the Football Association of Ireland (FAI), lobbied Varadkar (Irish Independent. Motion, 2012).

Alex White identified the strength of the alcohol industry in this debate:

The sports sponsorship was probably the trickiest of all because that's where a lot of the lobbying was done both from the industry and from the sports bodies, particularly the rugby and the soccer [organisations] (Interview C-2).

Varadkar had maintained that there was insufficient evidence that marketing or sponsorship restrictions would reduce under-age drinking, the key problem, in his view (Kelly, 2013). A cross-party group echoed Varadkar's concerns, saying that a ban would cause sporting organisations to “suffer inordinately” (Irish Examiner. Recommend, 2013). The nature of the opposition to the proposal, however, was interesting in that it varied across different dimensions of this bill. As White explained:

The remarkable thing [about the process] … was that nobody was against the Bill … everybody was for something being done … but different people opposed different parts of it and there was always somebody against some part of it (Interview C-2).

According to Ged Nash, there was also “resistance from senior figures in Fine Gael” and so there were growing concerns that the “public health approach … was going to be compromised” (Interview C-1). Labour cabinet ministers told the press about efforts by Fine Gael members to lobby them (Irish Independent. Plenty, 2012; de Breadun, 2012). In autumn 2013, cabinet dropped the sports sponsorship ban from the proposed bill (Danaher and Kelly, 2013). The media identified industry lobbying and Leo Varadkar's opposition as the key influences on that decision (Beesley, 2014).

In October 2013, the government released its alcohol strategy proposals. The legislation would comprise four main pillars: 1) minimum unit pricing; 2) the structural separation of alcohol from other products in shops; 3) restrictions on alcohol advertising and marketing and 4) health information on alcohol product and marketing, including health warnings and pregnancy advice. The plan represented the first time the government addressed alcohol as a “public health issue” (Irish Examiner, 2013) with sports sponsorship, likely important symbolically, omitted (McGee, 2013).

Despite the backing of the government, the alcohol legislation was slow to progress. A major cabinet shuffle in 2014 saw the legislation's two leading champions – Reilly and White – moved out of their portfolios, with Varadkar installed as the new Minister for Health.

Notwithstanding activities in his earlier ministerial brief, Varadkar took up the “legislation quite enthusiastically” and ended up doing “quite a lot of work on it”, White recalled (Interview C-2). In February 2015, the government published the General Heads of the Public Health (Alcohol) Bill and framed the bill as part of its broader *Healthy Ireland* framework (Collins, 2013). The bill outlined a goal of decreasing annual alcohol consumption from 11 to 9.1 L per capita by 2020 (Houses of the Oireachtas, 2015).

Varadkar's attention to alcohol harms and the potential role of population-level measures in curbing these harms dramatically shifted in his new position. Advocates described the then-health minister's medical background as conducive for policy learning. As one advocate explained:

The fact that [Varadkar's] a qualified medical doctor … really helped us because he understood [the details]. He just had to be briefed, he had to read the evidence, [and] read the papers to understand what was going on (Interview A-9).

Those with a long history with Varadkar described how his ideas about alcohol shifted over time. One interviewee reflected on a tense meeting with Varadkar in 2012 when he questioned the effectiveness of MUP and suggested that a ban on below-cost selling – a key alternative advocated by the alcohol industry – was an option. As that advocate explained:

If you think about the journey that [Varadkar] took from that [2012] meeting … it was just a phenomenal journey for him as a politician … accepting the evidence and … policy [from] the public health perspective as opposed to the industry perspective … he made a very significant, long, personal journey himself on this (Interview A-9).

Another interviewee articulated a similar experience.

A few years beforehand … we met with Leo Varadkar … about the sport sponsorship … he wasn't persuaded by our arguments … But it was possibly useful … given that he ended up subsequently as minister for health. When I met him again … we were on the same hymn sheet … it felt like we were on the same side (Interview A-8).

Varadkar's training and attention to the detail of the evidence available were important to his “journey” on alcohol. According to one former senator, Jillian van Turnhout:

[Leo Varadkar] had been on a journey … He's very evidence-focused … he can change his mind but only if the evidence is put to him … [I remember] at one of the hearings … I [asked if] he [would] consider putting cancer warnings [into the legislation], thinking he [would] say no. And [I remember] he came back and said, ‘yes, we will consider that’ (Interview C-3).

Others emphasised Varadkar's relationships with key figures in the health department, and specifically the CMO (Interview A-9). Two interviewees suggested that the bill would have been “watered down quite substantially” or would have had an entirely different fate if not for Varadkar (Interview B-2). In the words of one advocate:

The timing [of] getting a doctor coming into the Department of Health who listened to his chief medical officer [was critical] … we were very lucky … we could have had a minister there who just would have no interest and didn't want to know and [the bill] would never have happened then (Interview A-9).

As another advocate explained.

For me, the game-changer was Varadkar … public health legislation is legacy stuff … [and] the Taoiseach is the guy with the power. It was his influence that got [the bill] across the line (Interview A-4).

### 3.5 The opening of the policy window

In 2015, the government published the Public Health (Alcohol) Bill. Despite the introduction of the bill, there was little chance of it progressing before a forthcoming general election. The February 2016 election saw both Fine Gael and Labour lose seats, the latter heavily. The results ended the coalition government. Despite its electoral performance, Fine Gael retained power with a first ever confidence and supply agreement with Fianna Fail, its historic rival. The new government announced its programme in May 2016, which included a commitment “to enact the Public Health (Alcohol) Bill” (Programme for a Partnership Government: the Executive Summary, 2016). None of the major political parties opposed the bill.

The alcohol industry coalition functioned as the main opponent to the alcohol bill, using policy reports, press releases and op-eds to vocalise its opposition (MacMathuna, 2015; MacMathúna, 2016; DKM Economic Consultants, 2017). As other studies have noted, one of ABFI's key strategies was to reframe the debate, identifying alcohol “misuse” as the core problem to be addressed (Calnan et al., 2018).

According to interviews with elected officials (and confirmed with lobbying record data), most of the industry's lobbying efforts were led by trade associations, particularly the ABFI (Interviews C-1 and C-3). The industry coalition, however, was not entirely united in their opposition to the bill. As one expert explained:

The vintners, the publicans, which have a very good lobby group … came out in favour of minimum unit pricing in the hope that higher prices in the off-trade would lead people back into the on-trade (Interview B-1).

As such, industry actors focused on aspects of the bill where they could build the broadest coalition. Structural separation became a key target for industry lobbying. Retail trade associations claimed that the new regulations would burden small businesses with new construction and labour costs (O'Donovan, 2016). Interviewees described how effective the alcohol industry was in mobilising small businesses. As one expert explained:

My observation was that [the alcohol industry] had a very concerted campaign for working with the small convenience stores to submit their [concerns about the bill to the government] … But the big Tesco's and big international retailers aren't going to get the same sympathy from the public that the small local shop will (Interview B-1).

Another interviewee from the public health coalition described the effectiveness of this campaign:

We were blindsided by the ability of the industry and particularly the retailers to just mobilise their army of shopkeepers from around Ireland, who seemed to be all sent en masse to go running into their local politician … predicting calamity and disaster for their businesses (Interview A-8).

In October 2016, the legislative debate over the alcohol bill got underway in the Seanad (Senate, the upper house) (O'Donovan, 2016). Responsibility for progressing the legislation in the Seanad was left in the hands of a new junior health minister, Marcella Corcoran Kennedy (Fine Gael). The junior minister faced “enormous pressure from members of her own party” (Interviews A-8 and A-13). Fine Gael senators threatened to vote against the bill if the government failed to amend the structural separation provision (Ryan, 2016).

The alcohol industry's efforts to build a broader coalition of opponents to the structural separation was successful in slowing the legislative process down. As Ged Nash recalled:

Suddenly we had … people … managing small shops and local retail chains queuing outside our constituency offices telling us why the separation of alcohol … was going to be a huge expense (Interview C-1).

Broader political shifts prevented the alcohol bill from languishing in the upper house. In June 2017 Leo Varadkar replaced Enda Kenny as Fine Gael leader and Taoiseach (Prime Minister) after what was widely perceived as the disappointing general election performance. Varadkar appointed Simon Harris (Fine Gael) to serve as health minister and instructed him to progress the bill through both houses of parliament as soon as possible (O'Regan, 2017). As one advocate explained, “the fact that it was [Varadkar's] bill” before he became Taoiseach “certainly helped” (Interview A-9). Given ongoing industry opposition to the entire bill, as well as specific provisions within it, Varadkar could have chosen to focus on different priorities. As one former policy advisor explained:

Back in 2014 [Varadkar] could have stalled [the bill], he could have put the brakes on it but … he did the opposite … When he [later] became the leader of the country … he made it one of his priorities … Once he did that, it was game, set and match (Interview D-1).

Other interviewees stressed the CMO's skilful handling of the legislative process:

The Chief Medical Officer was very helpful … he's a tough wily political operator and his support was significant … whenever [the Bill] went before the Dáil or the Seanad … he spoke to it and he was there to answer the questions, so he very much owned the legislation (Interview A-4).

Although the bill required political leadership for enactment, the public health coalition was key in securing broader political support for the bill. Between 2016 and 2018, the Alcohol Health Alliance Ireland waged a highly sophisticated campaign to advance the legislation (Lesch and McCambridge, 2021b).

The chair of the Alcohol Health Alliance Ireland was Dr Frank Murray, a highly respected liver specialist who, at the time, had also been serving as the President of the RCPI. Interviewees described Murray as a calm, highly effective communicator who commanded a lot of respect (Interviews A-4, A-10, A-11, A-12, and B-2). His position at the RCPI also afforded advocates with key resources (Interview A-12).

According to one of the strategists behind the campaign:

We used to do a lot of briefing notes for [politicians], for [their] speeches in parliament … we would send them … ten points, ten facts that they could use. Things like ‘last week [this number of] ICU beds were all taken up by [alcohol-related admissions]’ … They took [the information] from us because we had credibility, we had authenticity … we were a trusted source of information (Interview A-3).

Minister Harris managed to forge a compromise with key senators, exempting smaller shops from the structural separation rules (Irish Examiner, 2017). To this end, interviewees credited the influence of Murray's political pragmatism, particularly when concessions had to be made to garner political support for the bill. As one politician explained:

Frank is … a very intelligent, clever operator in the best sense of that word. A very open player but very intelligent in the sense that he understands the process of compromise, he understands that … if you get three out of five things today, that's better than zero out of five (Interview C-2).

In September 2018, the alcohol industry and its allies waged a final lobbying campaign to oppose the bill in the lower house. Following meetings with officials and key opposition politicians, however, the health minister garnered enough support for the bill (McGee, 2018). In October 2018, after nearly 3 years of debate, and more than 6 years since the steering group's report, parliament passed the Public Health (Alcohol) Bill (O'Halloran, 2018). Since that time, health officials have been implementing different parts of the legislation. Some provisions took effect in 2019, including advertising restrictions and 2020, such as structural separation, whilst others have yet to commence, including MUP and product labelling).

## 4 Discussion and conclusion

Our study has drawn on interview data, policy documents and media coverage to deepen understanding of the decision to enact the Public Health (Alcohol) Bill in Ireland. Our findings illustrate how a series of interconnected developments, unfolding between 2008 and 2018, led to this historic policy decision.

First, we show how developments in the problem stream elevated the salience of alcohol-related harm in Ireland. The consequences of increased alcohol consumption became increasingly apparent to health experts in the late mid to late-2000s. Public concern over alcohol also reached new heights, as illustrated by the National Drugs Strategy's public consultations. The degree of public attention was also qualitatively different from earlier periods. Previously, there had been widespread recognition that alcohol consumption was on the rise (Hope and Butler, 2010). A decade later, however, the health and social impacts became much more apparent in public health data and across local communities.

Second, shifts in the institutional location of policymaking authority enabled public health advocates to reframe alcohol as a health issue. The creation of a steering group chaired by the CMO proved to be highly influential for the framing of the broad approach to alcohol policy and the specification of measures therein. The steering group had an explicit focus on, and mandate to, tackle the harms associated with alcohol. Previous attempts to develop recommendations in Ireland had broader remits and thus involved a much wider range of stakeholders (Butler, 2009; Hope and Butler, 2010). The venue shift in 2009 enabled the chair and his allies to frame the debate (i.e., alcohol as a public health problem) and generate specific policy recommendations (i.e., population-level measures).

Third, we show how the work of key actors, including politicians and public health advocates, helped generate and sustain political momentum for alcohol public health legislation. Two consecutive elections placed key individuals with an interest in alcohol policy into positions of influence. Several junior health ministers assumed responsibility for the legislation and helped clear several key institutional hurdles. Although Leo Varadkar had been an initial critic of the legislation, his appointment to Health Minister in 2015 and Taoiseach in 2017 proved vital to the progression of the bill. His efforts were supported and reinforced by the activities of a highly motivated and politically engaged public health advocacy coalition, the Alcohol Health Alliance Ireland. Previous observations have identified a lack of political leadership as a key impediment to legislative action (Butler, 2009, 2015). In this more recent period, advocates have been much better organised and the Department of Health has benefited from a string of strong and highly capable ministers keen to develop the application of the public health approach to alcohol-related harms in Ireland.

Our analysis clarifies the value of MSA as well as the ACF in explaining how alcohol policy unfolds. It has been observed that a major barrier to alcohol policy change in Ireland has been the tendency of politicians to “[pay] lip service to the views of public health experts” (Butler, 2015). Our analysis suggests that by the period under study, this was no longer possible for politicians due to the strengthening of health department institutional processes and leadership. Although the alcohol industry coalition used several tactics to undermine the bill's progression, its efforts were overwhelmed by other forces at play. The data sources limit the extent to which we may interrogate the conduct of the industry coalition. Our findings suggest that there was a gradual shift in Ireland, with the problem, policy and political streams eventually converging, and this window of opportunity being recognised by public health advocates, senior civil servants and political leaders. A powerful set of ideas (i.e., alcohol as a public health problem, population-level measures as the appropriate response) took shape in a novel institutional context (i.e., the CMO and Department of Health) and were subsequently championed by key cabinet ministers. Along with Frank Murray's leadership of public health advocates, this constellation of forces exerted enormous pressure in forming the wave that washed through the political system.

Proponents of the MSA often stress the role of policy entrepreneurs in fostering policy change (Kingdon, 1995; Zahariadis, 2003). Policy entrepreneurs can facilitate change by coupling the different streams at opportune moments. In this particular case, there was widespread agreement that several actors played distinct but complementary leadership roles. Across interviews and other key documents, Leo Varadkar, Tony Holohan, and Frank Murray emerged as the central players. Whilst all were key, Murray's behaviour appears most consistent with policy entrepreneurship. As noted across interviews, Murray exhibited political astuteness, detecting a shift in the national mood in 2015 and mobilising advocates at this key moment, just as Holohan had earlier recognised the need to gauge public support. Murray also demonstrated effective coalition management skills, keeping his allies on-side when the government watered down the bill.

Emerging research seeks to identify the specific attributes and skills that policy entrepreneurs bring to bear on policymaking (Mintrom and Norman, 2009; Anderson et al., 2020). The findings presented here might shed some light on this. It is noteworthy that Varadkar, Holohan, and Murray share a similar background, having all trained as medical doctors in the same city, though not all in the same medical school. It is possible in the context of a debate about health harms, individuals that come from medical backgrounds are perceived to be more credible and trustworthy. The size of the policy community in a small country such as Ireland may also be relevant here, as the individuals interviewed developed and reported on relationships with each other and with individuals even as they shifted into different roles.

The findings presented also hold implications for the broader study of alcohol policy. Other countries such as England have recently contemplated (Hawkins and McCambridge, 2019), or enacted population-level approaches to alcohol in Scotland and Wales (Lesch and McCambridge, 2020; Katikireddi et al., 2014b; Lesch and McCambridge, 2021c). These developments reveal some cross-national patterns. MUP legislation in Scotland was initially introduced by its Health Minister, Nicola Sturgeon. Similar to Varadkar's ascent, Sturgeon was then elected party leader and later elected as head of government. This parallel path to leadership via the management of the complex issues posed by alcohol legislation is noteworthy. Also similar to Scotland, in Ireland alcohol policy was a means by which the articulation of a distinctive new vision of health and society was given substance (McCambridge et al., 2014; Holden and Hawkins, 2013).

By contrast, in England, the UK government considered MUP but ultimately abandoned it in face of industry pressure. One difference is that within the UK government there was no clear policy champion prepared to see the policy change through to implementation (Hawkins and McCambridge, 2019). The policy context, viewed this way, is somewhat similar to Ireland prior to 2008. This suggests that the pursuit of population-level approaches to alcohol policy is unlikely to be successful in England in the absence of a well-placed policy champion or champions.

## Author contribution

Dr Matthew Lesch: Conceptualisation; Software; Validation; Methodology; Data curation; Investigation; Writing – original draft; Writing – review & editing; Project administration. Professor Jim McCambridge: Conceptualisation; Methodology; Validation; Funding acquisition; Writing – original draft; Writing – review & editing; Project administration; Supervision.

## Declaration of competing interest

None.
